# Medical and Physical Intelligence

**Published:** 1825-01

**Authors:** 


### MEDICAL AND PHYSICAL INTELLIGENCE.

### MISCELLANEOUS.

1. *Proceedings in the University of Edinburgh.*—It will be in the recollection of our readers, that, in the Number of the Journal for October last, we laid before them a sketch of the *proposed* new code of regulations for granting degrees in Medicine, in the University of Edinburgh. We stated at that time, that the said code was then in course of consideration; and we ventured to throw out a few hints for the assistance of the Senatus, in their decision on so important a subject. We have now to inform our readers, that the three last months of the year 1824 have been occupied in discussions growing out of the questions then agitated; but that, up to the moment at which we write (December 27th), it is not known in London that any positive decision has been come to. Here our communication would have ended, had we not lately been favoured with the perusal of some printed papers, by the Professor of Midwifery in that University, which contain matter much too curious and unexpected to be passed over in silence.

From these papers it appears that intestine war is now vigorously carried on among the Magnates of the Edinburgh College. The first blow appears to have been struck by the great body of Professors, who refused to make attendance on the midwifery class incumbent on such students as had already matriculated, or should matriculate prior to the 1st of January, 1825. Dr. Hamilton was, naturally enough, very wroth at a regulation which at once deprived him of perhaps five hundred pounds, independent of the *public* loss thereby sustained, and in that mood wrote an angry letter to the Principal, in which he declared his intention of appealing to the Town Council,—the patrons of the College. This led to a discussion as to the power of the Town Council, and we have it stated, as the opinion of some law-officers of eminence, that " their authority over the University appears to be more ample and unquestionable than the Senatus Academicus has, perhaps, hitherto been aware of."

Leaving the Senatus and their patrons to settle this knotty point, let us now inquire what has been the effect of admitting another member into the "Faculty of Physic." Dr. Christison has argued (with great good sense, as we think,) that, if a physician is to be taught midwifery, *nolens volens*, he ought, *a fortiori*, to be compelled to attend lectures on medical jurisprudence and police. To this (which appears to us a most legitimate principle,) no one, as far as we can discover, has any decided objection, except the Professor of Midwifery, who, having laboured for upwards of eight years to gain that seat in the Faculty of Physic which he has just acquired, turns round immediately upon his less fortunate brethren, and becomes in his turn a staunch monopolist. One of the letters before us is occupied with some harsh, but exceedingly clever, strictures on Dr. Christison's class, who had previously, it should be observed, "made some direct allusions, not quite respectful, to the class of Midwifery." But this is not all. The Faculty have actually projected three different curricula for the choice of the Senatus; in one of which they propose that Natural History should be added to the course of medical instruction. Mr. Russell, Professor of Clinical Surgery, upon this, asserts his claims, which he states to be sanctioned by the concurrence of the whole *practical* part of the profession. We suppose the Professors of Logic and Mathematics will come next;—any one, at least, has a better chance than the poor neglected Professor of Latin. Any study is considered as compatible with a medical education, except that of the dead languages.

The determination to have the examinations in English, seems to have undergone no revision. Dr. Hamilton thus expresses himself on the subject:—"On the utility of *this* change, it is quite unnecessary to offer any arguments. It will generally be admitted, in the present state of science, that the time spent in acquiring the habit of answering questions put in *Latin words*, might be better employed." Such are the opinions of a Professor who, in the very same breath, holds up the Universities of Cambridge and of Dublin as the models for Scotch imitation!—Here we were tempted to exclaim, "Oh! for one hour of that genius, now unhappily lost to the University of Edinburgh, whose pen, at the very first mention of such a proposition, would have started forward to vindicate the insulted majesty of the Latin language!" It is, indeed, but too obvious throughout the whole of the late proceedings of this University, that the author of the "Conspectus Medicinæ Theoreticæ" is no more!

From all that we have heard, we feel ourselves fully justified in saying, that the plan of examinations in the English language, is decidedly repugnant to the feelings of the profession throughout England. In this country, most happily, the love of classical literature still exists; nor will it be easy for the Faculty of Physic in Edinburgh to persuade their *English* students that one hour a-day, devoted to the study of Latin, (which, continued for four years, is far more than is really necessary to enable any man of moderate pretensions to pass a Latin examination with credit,) is time misspent. That they may succeed better with their Scotch pupils, we can more readily believe. What has amused us very much in this business, is Dr. Hamilton's holding up the example of America and of France as sanctioning this change; just as if French and American physicians were so much superior to those of England and Scotland, that we run the risk of losing our reputation, and perhaps our bread, unless we follow in their footsteps.

How or when all this is to end, we know not. But, as our readers must necessarily feel an interest in the result, we shall keep our eye on the subject, and revert to it on some future occasion.

*To the Editors of the London Medical and Physical Journal.*

Gentlemen,—By giving insertion to the following, you will greatly oblige A BOROUGH STUDENT.

*To* EDMUND BELFOUR, Esq. *Secretary to the Royal College of Surgeons.*

"Sir,—Being a student, at present prosecuting my studies in this metropolis, I beg to be informed, through the medium of this Journal, whether the law of the College of Surgeons, bearing date the 19th of March, 1824, has any reference to the young men who were apprenticed prior to the passing of that act?

"I consider, in justice to those whose indentures bear date prior to the 19th of March, 1824, that, when they go to the College for examination, that a diploma will not be refused them, merely because they have not complied with the late regulations."

*** Should Mr. Belfour think proper to give the information sought by our correspondent, we shall have much pleasure in giving it publicity.—EDITORS.

3. *Surgeon and Apothecaries' Drug Company.*—The Committee appointed to carry into effect the plan for establishing a National Benevolent Medical Fund, submit to the consideration of the profession the following Prospectus of a Company, to be entitled "The Surgeon and Apothecaries' Drug Company, and Benevolent Medical Institution."

The advantages contemplated by the Committee, in the success of this undertaking, are fourfold:—1stly, In the supply of *genuine* and *unmixed* Drugs and Chemicals; an object *utterly unattainable* under the present system,—2ndly, In a saving to the buyer of 20 per cent, on the prices hitherto established,—3rdly, In affording a certain and competent resource for the incapacitated practitioner, who has been unable to provide against the unfortunate contingencies of life,—And, 4thly, In securing to widows and orphans such a provision as shall place them above penury and distress, should every other resource be wanting. To effect these desirable objects the Committee submit the following propositions:—

1stly, To raise by shares of £25 each, a capital of £100,000. Subscribers to be practising Surgeons and Apothecaries; and shares to be transferable to practitioners only. An advance of 20 per cent. to be paid at the time of subscribing, and instalments of 20 per cent. to be called for as required; a guarantee being, however, given, that the whole of such instalments shall not be demanded within twelve months from the date of subscription.

2ndly, That no Subscriber be entitled, either for himself or his family, to the benefit of the Benevolent Fund, who holds less than *two* shares.

3rdly, That a dividend of 5 per cent. per annum on the capital advanced, be paid half-yearly to each Subscriber, and that the first profits of the Establishment be added to the Company's capital, until every original share of £25, amounts in value to the sum of £37. 10s. which will give to each subscriber an annual interest of 7½ per cent. on his capital actually advanced.

4thly, That as soon as the above increase in capital shall be effected (which the Committee calculate will be done in two years), all future profits to be appropriated to the Benevolent Fund, but no payment to be made to any claimant within three years of this time, unless the amount of the Benevolent Fund exceed the sum of £50,000.

5thly, That the *Benevolent Institution* consist of a Patron and Vice-Patrons, who, with the Directors of the Company for the time being, shall hold in trust the funds appropriated for this purpose.

6thly, To allow an annuity of £100 for life to every Subscriber who retires from business under *ill health or infirmity,* with a less income than £150 per year.

7thly, To allow to every widow (of Subscribers) the annual sum of £60 during her widowhood, provided she does not possess, from any other resource, an income exceeding the stipend from the Benevolent Fund; and to each child the annual sum of £10, until they attain the age of twenty-one years.

8thly, To grant to the widows and children of such Subscribers as have retired upon the benefits of the Benevolent Fund, the same provisions as the above.

9thly, That the value of the shares of every Subscriber, who retires or dies, be repaid to his representatives or assigns by the Company, unless he shall have participated, or his widow and children claim to participate, in the benefits of the Benevolent Fund; in either of which case, the shares of such Subscriber shall revert to the Company without fee or purchase.

10thly, In the case of transfer of shares, no benefit to accrue, either to the purchaser or his family, until five years from the date of such transfer.

11thly, That such professional gentlemen who do not practise pharmacy, be invited to support the Institution, by paying an annual sum of two guineas, which will entitle them and their families (if needful) to the benefit of the Benevolent Fund.

Communications to be addressed to the Committee, at Mr. J. Churchill's, Bookseller, Saville House, Leicester-square, where books are opened to receive the names of subscribers.

### BILLS OF MORTALITY,

*From December* 17, 1823, *to December* 14, 1824.

| DISEASES. | |
|---|---|
| Abscess | 96 |
| Age and Debility | 1369 |
| Apoplexy | 333 |
| Asthma | 716 |
| Bile | 2 |
| Cancer | 98 |
| Childbed | 169 |
| Cholera Morbus | 2 |
| Consumption | 4980 |
| Convulsions | 2772 |
| Croup | 94 |
| Diabetes | 1 |
| Diarrhœa | 5 |
| Dropsy | 780 |
| Dropsy in the Brain | 762 |
| Dropsy in the Chest | 73 |
| Dysentery | 2 |
| Enlargement of the Heart | 9 |
| Epilepsy | 45 |
| Eruptive Diseases | 27 |
| Erysipelas | 14 |
| Fever | 750 |
| Fever (Typhus) | 37 |
| Fever, Intermittent, or Ague | 2 |
| Fistula | 5 |
| Flux | 2 |
| Gout | 20 |
| Hæmorrhage | 31 |
| Hernia | 33 |
| Hooping-Cough | 627 |
| Hydrophobia | 7 |
| Inflammation | 2116 |
| Inflammation of the Liver | 137 |
| Insanity | 144 |
| Jaundice | 26 |
| Jaw-locked | 2 |
| Measles | 966 |
| Miscarriage | 3 |
| Mortification | 232 |
| Palsy | 144 |
| Rheumatism | 8 |
| Scrofula | 14 |
| Small-pox | 725 |
| Sore Throat or Quinsey | 13 |
| Spasm | 52 |
| Still-born | 824 |
| Stone | 20 |
| Stoppage in the Stomach | 18 |
| Suddenly | 104 |
| Teething | 388 |
| Thrush | 65 |
| Tumor | 10 |
| Venereal | 4 |
| Worms | 4 |
| Total of Diseases | 19882 |

| CASUALTIES. | |
|---|---|
| Bruised | 1 |
| Burnt | 30 |
| Choked | 1 |
| Drowned | 149 |
| Excessive Drinking | 5 |
| Executed* | 5 |
| Found Dead | 5 |
| Fractured | 1 |
| Frightened | 1 |
| Killed by Falls and several other Accidents | 86 |
| Murdered | 1 |
| Ossification of the Heart | 1 |
| Poisoned | 4 |
| Scalded | 4 |
| Shot | 1 |
| Smothered | 1 |
| Starved | 1 |
| Suffocated | 5 |
| Suicides | 52 |
| Total of Casualties | 355 |

| CHRISTENED. | | | BURIED. | | |
|---|---|---|---|---|---|
| Males | 12978 | In all, 25758 | Males | 10565 | In all, 20237 |
| Females | 12780 | | Females | 9672 | |

Whereof have died,

| | | | |
|---|---|---|---|
| Under Two Years of Age | 6476 | Fifty and Sixty | 1742 |
| Between Two and Five | 2103 | Sixty and Seventy | 1715 |
| Five and Ten | 798 | Seventy and Eighty | 1411 |
| Ten and Twenty | 764 | Eighty and Ninety | 593 |
| Twenty and Thirty | 1296 | Ninety and a Hundred | 84 |
| Thirty and Forty | 1444 | A Hundred and Three | 1 |
| Forty and Fifty | 1809 | A Hundred and Seven | 1 |

Decreased in the Burials this Year, 350.

* There have been executed within the Bills of Mortality, 10; only 5 have been reported as such.

### METEOROLOGICAL JOURNAL,

*From November* 20, *to December* 19, 1824.

By Messrs. William Harris and Co. 50, Holborn, London.

| | MOON. | Rain gauge | THERM. | | | BAROM. | | DE LUC's HYG. | | WIND. | | ATMOSPHERIC VARIATIONS. | | |
|---|---|---|---|---|---|---|---|---|---|---|---|---|---|---|
| | | | 9 A.M. | MAX. | MIN. | 9 A.M. | 10P.M. | 9 A.M. | 10P.M. | 9 A.M. | 10P.M. | 9 A.M. | 2 P.M. | 10P.M. |
| Nov | | | | | | | | | | | | | | |
| 20 | ● | .48 | 50 | 53 | 56 | 29.47 | 29.36 | 97 | 90 | E | SW | Rain | Rain | Fine |
| 21 | | | 58 | 59 | 42 | 29.36 | 29.30 | 85 | 89 | W | W | Fine | | Rain |
| 22 | | | 43 | 47 | 42 | 29.30 | 29.19 | 86 | 84 | SSW | SSE | | Fine | |
| 23 | | .59 | 52 | 55 | 37 | 28.50 | 28.58 | 75 | 75 | S | SW | Cloud. | Rain | Fine |
| 24 | | | 46 | 50 | 42 | 28.91 | 29.05 | 76 | 78 | SW | SW | Fine | Fine | |
| 25 | | .47 | 44 | 48 | 35 | 29.28 | 29.42 | 83 | 83 | WSW | WNW | | | |
| 26 | | | 36 | 45 | 37 | 29.57 | 29.62 | 80 | 80 | WNW | N | Foggy | | |
| 27 | | | 42 | 44 | 42 | 29.63 | 29.73 | 75 | 75 | ENE | SE | | | Cloud. |
| 28 | ☽ | | 52 | 54 | 46 | 29.60 | 29.42 | 90 | 80 | SW | SW | Rain | Cloud. | |
| 29 | | | 51 | 52 | 42 | 29.13 | 29.44 | 75 | 80 | WSW | WSW | Fine | Fine | Fine |
| 30 | | | 47 | 48 | 42 | 29.41 | 29.15 | 82 | 90 | SW | SSW | Cloud. | Rain | Rain |
| Dec | | | | | | | | | | | | | | |
| 1 | | .31 | 42 | 42 | 35 | 29.23 | 29.42 | 75 | 80 | W | WSW | | Fine | Fine |
| 2 | | | 39 | 47 | 37 | 29.54 | 29.24 | 81 | 85 | WSW | SW | Foggy | Rain | Rain |
| 3 | | 50 | 39 | 45 | 36 | 29.35 | 29.60 | 82 | 78 | NW | WSW | Fine | Fine | Fine |
| 4 | | | 42 | 47 | 37 | 29.28 | 29.30 | 85 | 90 | NE | WSW | Cloud. | Rain | Rain |
| 5 | | | 39 | 40 | 35 | 29.56 | 29.90 | 95 | 92 | N | N | | Fine | Fine |
| 6 | ○ | | 39 | 44 | 37 | 29.74 | 29.46 | 78 | 82 | W | SW | Foggy | | Rain |
| 7 | | .17 | 41 | 43 | 36 | 29.42 | 29.59 | 87 | 83 | W | WNW | Cloud. | | Fine |
| 8 | | .11 | 40 | 45 | 39 | 29.76 | 29.66 | 87 | 91 | WSW | WSW | Sl. Fog | Fine | Rain |
| 9 | | .15 | 40 | 42 | 33 | 29.63 | 29.52 | 95 | 92 | WSW | WSW | Fog | | |
| 10 | | | 35 | 44 | 34 | 29.77 | 30.03 | 86 | 88 | W | WSW | | | Fine |
| 11 | | | 44 | 46 | 45 | 30.00 | 30.06 | 95 | 100 | WSW | WSW | | Sleet | Cloud. |
| 12 | | | 49 | 50 | 46 | 30.20 | 30.30 | 94 | 94 | W | W | Fine | Fine | |
| 13 | ☾ | | 48 | 47 | 45 | 30.27 | 30.34 | 96 | 91 | WSW | W | | | Fine |
| 14 | | | 46 | 47 | 44 | 30.35 | 30.24 | 98 | 100 | W | WSW | Foggy | Foggy | Cloud. |
| 15 | | | 47 | 48 | 44 | 30.00 | 29.83 | 98 | 93 | WSW | W | Cloud. | Cloud. | Cloud. |
| 16 | | | 45 | 51 | 38 | 29.83 | 29.90 | 86 | 85 | N | WNW | Fine | Fine | Fine |
| 17 | | | 41 | 44 | 42 | 29.93 | 29.96 | 90 | 96 | W | WSW | | Sho'ry | Sleet |
| 18 | | | 43 | 49 | 45 | 30.05 | 30.06 | 98 | 92 | E | WSW | Cloud. | Cloud. | Cloud |
| 19 | | .39 | 50 | 52 | 48 | 29.98 | 29.80 | 94 | 91 | W | SW | | | |

The quantity of rain fallen in the month of November, was 4 inches and 18.100ths.

